# Photoinduced Negative Differential Resistivity and Gunn Oscillations in SrTiO3

**DOI:** 10.1002/advs.202306420

**Published:** 2023-10-23

**Authors:** Mehrzad Soleimany, Marin Alexe

**Affiliations:** ^1^ Department of Physics University of Warwick Gibbet Hill Road Coventry CV4 7AL UK; ^2^ Department of Materials and Earth Sciences Technical University of Darmstadt 64287 Darmstadt Germany

**Keywords:** Gunn oscillation, negative differential resistance, photo‐conductivity, transferred‐electron devices

## Abstract

SrTiO_3_, a perovskite oxide, holds significant potential for application in the field of oxide electronics. Notably, its photoelectric activity in the low temperature regime, which overlaps with the quantum paraelectric state, exhibits remarkable characteristics. In this study, it is demonstrated that when photo‐excited with above band gap energy photons, SrTiO_3_ exhibits non‐linear transport of photocarriers and voltage‐controlled negative resistance, resulting from an intervalley transfer of photo‐induced electrons. As a consequence of the negative resistance, the photocurrent becomes unstable and spontaneously gives rise to low frequency Gunn‐like oscillations.

## Graphical abstract

SrTiO_3_ crystals exhibit non‐linear transport of photo‐carriers excited with above band photons, generating a voltage‐controlled negative resistance stemming from an intervalley transfer of photo‐induced electrons. As a consequence of the negative resistance, the photocurrent becomes unstable and spontaneously gives rise to low frequency Gunn‐like oscillations.

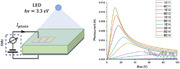

## 1 Introduction

SrTiO_3_ (STO) is a wide band gap semiconductor displaying a plethora of effects that include metallic‐like conductivity by n‐type doping, high electron mobility exceeding 2 · 10^4^ cm^2^ V^−1^ s^−1^,^[^ ^1^ ^]^ superconductivity at 0.28 K,^[^ ^2^ ^]^ large Seebeck coefficient,^[^ ^3^ ^]^ quantum paraelectric state under 37 K,^[^ ^4^ ^]^ and room temperature ferroelectricity under compressive strain.^[^ ^5^ ^]^ STO has also remarkable photoelectric activity featuring anomalous photoconductivity,^[^ ^6^ ^]^ and photoluminescence,^[^ ^7^ ^]^ or coherent photo‐electron emission.^[^ ^8^ ^]^

Gunn effect has been discovered at the eve of semiconductor technology by J. B. Gunn of IBM in 1963.^[^ ^9^ ^]^ It has been revealed that certain semiconductors exhibit a complex band structure characterized by the presence of at least two valleys in the conduction band. The primary valley exhibits high electron mobility, while an upper satellite valley has lower mobility. Under normal conditions with low applied fields, the semiconductor behaves in an ohmic manner. However, at high applied fields, the electrons gain enough energy to occupy levels in the satellite valley with lower mobility. This phenomenon, known as the transferred‐electron effect, occurs once the applied field exceeds a specific threshold. As a consequence, the semiconductor enters a regime of differential negative resistance. This leads to subsequent instabilities and oscillations in the flowing current. This effect has triggered flourishing research on instabilities in solid‐state electron devices as well as developing semiconductor devices such as Gunn, IMPATT, or TRAPATT diodes with direct application in microwave technology.^[^ ^10^, ^11^ ^]^

We show here that photoinduced currents in STO at low temperatures in the quantum paraelectric state exhibit transferred‐electron effect, characterized by negative differential resistance and Gunn oscillations, albeit at low frequencies. This adds a new dimension to the existing complexity of electronic properties of STO in which not the thermally excited carriers, but the photoexcited carriers are generating non‐linear electron transport and instabilities associated with local mobile/moving polar regions and instabilities. This is not a simple resembling to the semiconductors with high mobility such as GaAs, since STO is by far richer offering in this quantum paraelectric regime a complex electronic behavior extending from semiconducting to metallic and superconducting phases all separated by marginal variations in carrier energy and temperature.^[^ ^12^ ^]^ The present findings underscore the unique nature of STO as a perovskite oxide with a complex electronic band structure and remarkably high mobility of photoinduced carriers.

## 2 Experimental Results and Discussions

Photoconductivity of a nominally pure SrTiO_3_ (STO) (100)‐oriented single crystal has been investigated at low temperatures (<50 K) under uniform illumination with monochromatic light with λ  =  375 nm. The wavelength has been chosen in such way that the photon energy (*h*ν  =  3.3 eV) shall be as close as possible to maximum of the spectral response of STO, which is slightly larger than the STO band gap (3.25 eV) and generate free carriers by band‐band excitation.^[^ ^6^ ^]^

**Figure 1:**
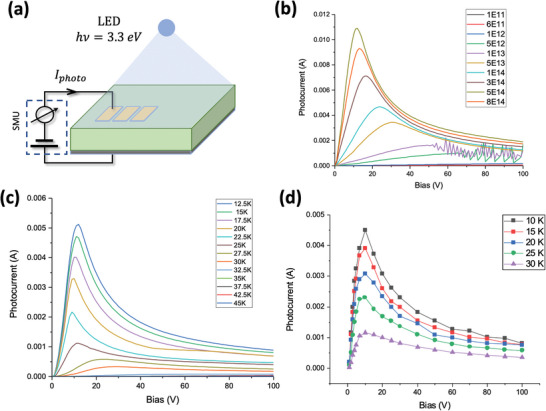
a) Schematic of sample and the photoconductivity measurement setup; b) out‐of‐plane (OOP) photoconductive current as a function of applied voltages at different incident photon fluxes measured at 8 K, and c) photoconductive current as a function of applied voltages at different temperatures; d) Photoconductive current from pulse measurements. Temperature‐dependent measurements were performed under illumination with 3 · 10^14^ cm^−2^ s^−1^ photon flux.

High‐quality crystal has been prepared as described in Experimental Section to produce atomically flat vicinal surface. Top semi‐transparent gold electrodes have been deposited on this surface.^[^ ^13^ ^]^ The photoconductive properties have been investigated by measuring the current‐voltage (IV) characteristics, respectively the short‐circuit photocurrent at different applied voltages, at different temperatures and under variable photon flux in the range of (10^11^ − 10^15^) cm^−2^ s^−1^ for both out‐of‐plane (OOP), i.e., between Au electrodes and bottom silver contact, and in‐plane (IP) geometries. A typical photo‐IV characteristics family under different photon flux is shown in **Figure** **1**b. The photo‐IV characteristics are highly non‐linear showing three main features: a distinctive peak, a region of negative differential resistance, and a strong instability of the photocurrent in certain range of the incident flux and applied field.

The photocurrent rises very fast with the applied field up to a critical value beyond which the photocurrent quenches rapidly, generating a current peak and an extended negative differential resistivity (NDR) regime. The field and photocurrent peak values are both temperature and photon flux dependent, as can be seen in Figure 1b,c. Above 45 K the photocurrent is few orders of magnitude lower and behaves “normally” without any peak and associated NDR regime.

Given very high values of the current flowing through the sample, there is a motivated suspicion that the field‐induced current quenching in NDR regime occurs because of a Joule effect, i.e., local heating. In order to rule out such Joule effect, the current‐voltage characteristics have been raised using pulsed measurements (see Figures S1 and S2, Supporting Information). The results, shown in Figure 1d, are very similar to the dc measurements, pointing that a local heating, if any, has only a marginal effect on electron transport and, obviously, is not at the origin of the NDR effect.

NDR effects are usually generated by two main mechanisms generating two different shapes of the IV characteristics. The S‐shape or the current‐controlled NDR is generally based on impact ionization, filamentary conduction generated by the local phase change or soft breakdown.^[^ ^14^ ^]^

The IV characteristics shown here belong to the second type, the voltage controlled negative resistance, generating an N‐type IV characteristics.^[^ ^15^ ^]^ This type of NDR is usually produced in classical bulk semiconductors by a transferred‐electron effect or in special quantum tunnel devices such as Esaki or resonant tunneling diodes.^[^ ^16^, ^17^ ^]^ In the present case the device is pure bulk, thus the type is the voltage controlled NDR generated by a transferred‐electron effect. The mechanism behind, called Ridley–Watkins mechanism,^[^ ^18^ ^]^ is characterized by transfer of free electrons from the high mobility energy valley of the energy band structure to a low‐mobility, higher energy satellite valley.^[^ ^19^ ^]^ The onset conditions of such electron transfer mechanism and the consequent NDR are: i) a low lattice temperature, ii) a high mobility in the lower conduction‐band minimum, and iii) a band structure showing an upper satellite valley with low mobility and high density of available states.^[^ ^14^ ^]^ In the STO case the conditions might be well fulfilled. The effect occurs at temperatures under 40 K where STO enters the quantum paraelectric state characterized not only by very high dielectric permittivity but also by high mobility of free carriers. The photoconduction is primarily n‐type with photo‐excited electrons having a mobility exceeding 10^4^ cm^2^ V^−1^ s^−1^ at 4 K, as shown by photo‐Hall mobility measurements.^[^ ^20^ ^]^ The only remaining condition to fulfil is the existence of a satellite valley with a lower mobility. As it is known, STO is a non‐trivial band semiconductor with a band structure affected by atom and Rashba spin–orbit coupling, as shown by recent magneto‐transport investigations.^[^ ^21^ ^]^ A band splitting of about 0.3 eV has been theoretically predicted and experimentally observed.^[^ ^22^, ^23^ ^]^ Additional experimental evidence on two band transport with two mobilities of 3 and 1200 cm^2^ V^−1^ s^−1^ has been given by Guduru et al.^[^ ^24^ ^]^

So, we can assume that minimum conditions for occurrence of a transferred‐electron effect are fulfilled for STO at low temperatures. We can then expect that in the low field regime, most of the free electrons are in the lower conduction‐band minimum (high mobility) showing a quasi‐ohmic behavior. Under increasing applied field, the electrons drift velocity will approach the thermal velocity entering non‐linear regime featuring hot carriers. If the electron gas equivalent thermal energy *kT*_e_ is higher than the difference between the valley minima Δ*E*, i.e., *kT*_e_ > Δ*E*, there will be certain probability of electrons to transfer and occupy states in the upper valley (lower mobility). Fingerprints of such electron transfer mechanism is a clear threshold in applied field for the onset of the negative differential resistivity. Since the STO band structure is in the first approximation temperature independent, the threshold field shall be temperature independent. This can be seen in Figure 1d where the onset of the NDR is at the same applied voltage irrespective of the temperature.

**Figure 2:**
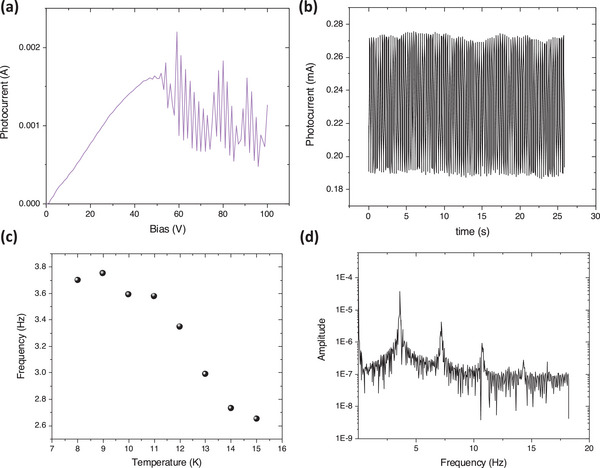
a) OOP IV characteristic measured at 8 K under 10^13^ cm^−2 ^s^−1^; b) time dependence of the photocurrent at 10 K under illumination with 375 nm with photon flux of 10^13^ cm^−2 ^s^−1^ and 50 V applied voltage; c) temperature dependence of the fundamental frequency determined from Fourier analysis of the time‐dependent photocurrent; d) Fourier analysis of the oscillating photocurrent in (b).

**Figure 3:**
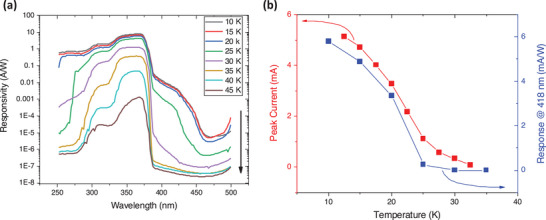
a) Spectral distribution of the photo‐response, i.e., the photoconductive current at 0.5 V applied bias normalized to the spectral power density, measured at different temperatures; b) Peak values of the photocurrent from Figure 1c and the photo‐response measured at 418 nm (2.97 eV) from (a) as a function of temperature.

An important feature which is related to the transferred‐electron effect and the associated NDR effect is a general instability which triggers an oscillating current flowing through the device. These oscillations were initially observed on GaAs and subsequently named Gunn oscillation, after the author of the pioneering report.^[^ ^9^ ^]^ In the GaAs, soon after the electric field is applied and the systems enters the NDR regime, the current starts oscillating. Special conditions in terms of sample geometry, applied field, carrier density/doping, and/or low temperature are needed to stimulate this effect. Here in the STO case, the oscillations occur only in photo‐excited crystals with a certain photon flux situated in a relatively narrow window, respectively between 10^12^ and 10^13^ cm^−2^ s^−1^. The applied field shall be sufficiently large, i.e., about 1 kV cm^−1^, to bring the system in the NDR regime (see **Figure** **2**a). Soon after applied flux‐field conditions are fulfilled, quasi‐sinusoidal oscillations are self‐sustaining without changing the fundamental frequency, as shown **Figure** **3**. The amplitude is not constant due to superposition of higher harmonics revealed by Fourier analysis shown in Figure 2d and an additional chaotic behavior which is typical for such instabilities.^[^ ^11^ ^]^ While sinusoidal oscillations are detected in the optimum conditions, approaching the boundary conditions in terms of incident flux and applied field led to instability in oscillations, even showing chaotic behavior, as in the case of classical semiconductors.^[^ ^25^ ^]^

Similar IV characteristics, photocurrent instabilities and oscillations have been measured on the in‐plane geometry, as shown in Figure S3 (Supporting Information). In this geometry the oscillations are having a more chaotic behavior with a clear fundamental frequency of about 5.5 Hz. This chaotic or multifrequency behavior is mostly due to an inhomogeneous field distribution and undefined device length.

The Gunn effect has been explained by an inhomogeneous carrier distribution across the crystal and occurrence of space‐charge domains of lower and higher fields related to accumulation and depletion layers.^[^ ^26^ ^]^ These domains are nucleating at one electrode, usually cathode, and travel across the crystal similar to a wave to disappear at the other electrode. Oscillations will occur when proper conditions related to the nucleation and travel time of these domains are achieved. Generally, the oscillations frequency is determined by the intrinsic relaxation time τ_r_, which is the characteristic time the system would recover from a perturbation of the charge distribution, such as these moving high‐low field domains. The space charge domains will form and disappear in a time order of few times the dielectric time constant associated with the slope of the resistance in the NDR regime. The relaxation time constant, defined as the product between the dielectric permittivity and resistivity, i.e., τ_r_ = ε_r_ε_0_ρ, is for GaAs and similar classical semiconductors very small, the frequency of the generated oscillations being in the 10^9^ Hz range. In the present STO case the frequencies are low, only few Hz, as can be inferred from Figure 2. The intrinsic relaxation time constant of STO is very high, since the investigated crystal is non‐doped, and the relative dielectric permittivity is extremely high. It reaches values above 10^4^ because STO enters the quantum paraelectric state under 37.5 K. With an estimated resistivity in the NDR regime of about 3 · 10^4^ Ω and measured capacitance of 205 pF the relaxation time constant shall be in the order of 10^−6 ^s, giving an equivalent frequency much higher than the observed oscillations.

As it has been shown, the existence of at least one impurity level in the band gap interacting with the two non‐equivalent valleys in the conduction band can decrease the frequency of the Gunn oscillations.^[^ ^27^,^28^ ^]^ A critical factor in slowing down the oscillation is a relatively high trapping probability of electrons from both valleys by the impurity level. To slow down the oscillation, the transfer rate from the trap to the valleys should be lower compared to the inter‐valleys transfer rates. Similar to the classical case of Gunn oscillation, the formation and propagation of space‐charge domains are observed in this scenario. The velocity of domain propagation, denoted as *u*, is related to the drift velocity, denoted as *v*, by the following relationship:

(1) $$u\;=\;v\;\left(\tau_{t}/\tau_{g}\right)/\left(1-\tau_{t}/\tau_{k0}\right)$$

where τ_g_ is the average capture time on the trap related to reciprocal transition rate to and from the impurity level to both valleys, τ_t_ is a response time related to inter‐valley transition rates and τ_g_, and τ_k0_ is the dielectric relaxation time in the NDR regime. Further on, τ_t_/τ_g_ = *n*_w0_/*N*, where *n*_0_ is the steady‐state density of electrons in the valleys and *N* is the total density of electrons free and trapped.^[^ ^27^ ^]^ Finally in the condition that the dielectric relaxation time is much larger than τ_t_ the domain propagation velocity *u* is simply:

(2) $$u\;=\;v\frac{n_{0}}{N}$$

In the present case the density of free electrons *N* can be estimated assuming that at these low temperatures there are no thermally excited free carriers but only photoinduced. The upper limit of the carrier density can be taken as the incoming light flux (10^13^ cm^−2^) divided by the crystal thickness (0.05 cm). The density of electrons in the valleys *n*_0_ is totally unknown for STO, but it can be assumed being in the order of magnitude of 10^7^ cm^−3^ as in ref.[27]. The drift velocity *v*  =  µE calculated with 10^4^ cm^2 ^V^−1^ s^−2^ electron high mobility and *E* given by the NDR onset voltage (see Figure 2a) is the order of 10^7^  cm s^−1^. In these conditions, the domain velocity *u* is the order of 0.5 cm s^−1^ and the transit time of the Gunn domains would be 0.1 s. Thus, the equivalent frequency would be 10 Hz, fitting relatively well with the experimental value of about 3.5 Hz. However, decreasing the sample thickness, will slightly increase the fundamental frequency, as shown in Figure S4 (Supporting Information). For 100 µm thick crystal, the fundamental frequency is 5.5 Hz, similar to the oscillations detected in the IP configuration, confirming thus the device length dependence.

It is clear that the trap levels do play a major role and practically define the Gunn oscillations in STO. It is therefore sensible to verify if there are photoelectrically active trap levels in STO that could potentially play a role in the Gunn oscillations. We have thus investigated the photoconductive current at different wavelengths between 500 and 250 nm. The resulting spectral distribution shown in Figure 3 reveals a significant photo‐response in the 400 nm to 450 nm range resembling a broad peak centered at about 420 nm. The notable observed sub‐band activity is associated with levels distributed between 3.1 and 2.75 eV, likely aggregated into a sub‐band structure. Interestingly, the response of these levels exhibits a significant decrease with increasing temperature and is only active below 35 K. This suggests a potential connection between these sub‐band levels and the occurrence of the oscillations. Figure 3b further illustrates this relationship by depicting the temperature dependence of the sub‐band activity alongside the peak value of the photo‐IV characteristics shown in Figure 1c. Remarkably, both plots display a similar behavior, with a significant decrease in activity above 25 K, following a sigmoidal pattern. While we cannot establish a direct causal relationship between these sub‐band levels and the onset of negative differential resistance and Gunn oscillations, the nearly identical temperature dependence of these two parameters strongly suggests a link between them.

## 3 Conclusions

We have shown here that the photocurrent induced by photons with above band gap energy of 3.3 eV (λ  =  375 nm) in a nominally undoped SrTiO_3_ behaves abnormally with the applied electric field. Under illumination with an incident flux higher than about 5 · 10^12^ cm^−2^ s^−1^ and at temperatures under 40 K the current–voltage characteristics becomes non‐linear showing a voltage‐controlled negative resistance regime. This is most probably generated by an intervalley transfer of photo‐induced electrons similar to Ridley and Walking mechanism.^[^ ^17^ ^]^ As in the case of classical semiconductors, soon the system enters the NDR regime the photocurrent becomes instable generating Gunn‐like oscillations. The oscillation frequencies are rather low due to existence of at least one active impurity level within the band gap which limits the velocity of the Gunn domains. Overall, the non‐linear transport of photocarriers, negative differential resistance, and photo‐induced Gunn effect point to the fact that SrTiO_3_ is closer to the classical semiconductor behavior than any other perovskite oxide. While this effect exhibits many similarities to the classical high mobility semiconductors, such as GaAs, in SrTiO_3_ it presents a far more complex electronic behavior, particularly within the quantum paraelectric regime. Unlike typical semiconductors, SrTiO_3_ offers a remarkably rich phase diagram extending from metal, semiconductor to superconducting phases all separated by marginal variations in carrier energy and temperature.^[^ ^12^ ^]^ The noteworthy aspect of the present findings is that non‐linear electron transport and instabilities associated with local polar regions are not generated by thermally excited carriers, as is often the case in semiconductors, but by photoexcited carriers, adding a new dimension to the already complex phase diagram of SrTiO_3_.

Further understanding of the band structure and a search for other donor doping elements than classical La and Nb might enable new opportunities for exploration and innovation in the field of oxide electronics.

## 4 Experimental Section

In this study nominally pure (001)‐oriented SrTiO_3_ crystals 0.1^○^ mis‐cut against the (110) crystallographic direction [Crystec GmbH] have been used. The initial 5 × 10 mm^2^ crystal has been cut in 5 × 5 mm^2^. Special care has been taken to ensure high‐quality STO surface to prevent high surface carrier recombination rate. Following a basic cleaning process, the polished surface has been etched in buffered HF and annealed/recrystallized at 1100 ^○^C for an hour to produce atomically flat, vicinal surface. Sample surface morphology was measured by atomic force miscroscopy in tapping mode using a Bruker Icon microscope.

For electric characterization three semi‐transparent Au electrodes (<10 nm thick) 1  ×  0.5 mm^2^ with a gap of 100 µm between them have been sputtered on the vicinal surface (see Figure 1a). Photoelectric measurements at low temperatures (8—50 K) have been performed by mounting the sample with silver paste in a dry cryostat (attoDRY 800, attoCube) provided with a fused silica ultraviolet (UV) transparent window. Sample has been illuminated with a uniform UV light (λ  =  375 nm) generated by a UV light‐emitting diode (LED375L, Thorlabs). The photo flux has been measured at the cryostat window using a calibrated Si photodiode (S1337, Hamamatsu). The current has been measured using a source measuring unit Keithley 2535 either between the top‐electrode and bottom silver electrode (OOP configuration) or between the top Au electrodes (IP configuration).

Time‐dependent measurements were performed in classical photoconductive mode with a 120 Ω load resistance serially connected with the sample and a constant voltage source connected on both (see Figure S1, Supporting Information). The output signal sampled as a voltage signal on the load resistance was visualized and digitalized with an oscilloscope (Agilent, DSO‐3032).

The spectral distribution of the photo‐current was measured using a 100 W Xe‐lamp broadband light source and a monochromator. The spectral power of the light source was measured at the cryostat entrance using the same calibrated Si photodiode. The spectral distribution of photo‐response of the STO crystal has been calculated by normalizing the photocurrent measured at 0.5 V applied bias to the spectral power of the lamp.

## Conflict of Interest

The authors declare no conflict of interest.

## Supporting information

Supporting Information

## Data Availability

The data that support the findings of this study are available from the corresponding author upon reasonable request.

## References

[1] O. N. Tufte , P. W. Chapman , Phys. Rev. 1967, 155, 796.

[2] J. F. Schooley , W. R. Hosler , M. L. Cohen , Phys. Rev. Lett. 1964, 12, 474.

[3] T. A. Cain , A. P. Kajdos , S. Stemmer , Appl. Phys. Lett. 2013, 102, 182101.

[4] K. A. Müller , H. Burkard , Phys. Rev. B 1979, 19, 3593.

[5] D. Lee , H. Lu , Y. Gu , S.‐Y. Choi , S.‐D. Li , S. Ryu , T. R. Paudel , K. Song , E. Mikheev , S. Lee , S. Stemmer , D. A. Tenne , S. H. Oh , E. Y. Tsymbal , X. Wu , L.‐Q. Chen , A. Gruverman , C. B. Eom , Science 2015, 349, 1314. 26383947 10.1126/science.aaa6442

[6] T. Feng , Phys. Rev. B 1982, 25, 627.

[7] D. Kan , T. Terashima , R. Kanda , A. Masuno , K. Tanaka , S. Chu , H. Kan , A. Ishizumi , Y. Kanemitsu , Y. Shimakawa , M. Takano , Nat. Mater. 2005, 4, 816.

[8] C. Hong , W. Zou , P. Ran , K. Tanaka , M. Matzelle , W.‐C. Chiu , R. S. Markiewicz , B. Barbiellini , C. Zheng , S. Li , A. Bansil , R.‐H. He , Nature 2023, 617, 493. 36889355 10.1038/s41586-023-05900-4

[9] J. B. Gunn , Solid State Commun. 1963, 1, 88.

[10] P. J. Bulman , G. S. Hobson , B. C. Taylor , Transferred Electron Devices, Academic Press, London‐New York 1972.

[11] H. L. Grubin , V. V. Mitin , E. Schöll , M. P. Shaw , The Physics of Instabilities in Solid State Electron Devices, Plenum Press, New York 1992.

[12] S. E. Rowley , L. J. Spalek , R. P. Smith , M. P. Dean , M. Itoh , J. F. Scott , G. G. Lonzarich , S. S. Saxena , Nat. Phys. 2014, 10, 367.

[13] R. H. Cornely , N. Fuschillo , J. Vac. Sci. Technol., B: Nanotechnol. Microelectron.: Mater., Process., Meas., Phenom. 1974, 11, 163.

[14] M. Salverda , R. P. Hamming‐Green , B. Noheda , J. Phys. D: Appl. Phys. 2022, 55, 335305.

[15] S. M. Sze , Y. Li , K. K. Ng , Physics of Semiconductor Devices, Wiley, Hoboken, NJ, USA, 2021.

[16] L. Esaki , Phys. Rev. 1958, 109, 603.

[17] C. Song , H. Mao , Y. Yang , X. Liu , Z. Yin , Z. Hu , K. Wu , J. Zhang , Adv. Funct. Mater. 2022, 32, 2105256.

[18] B. K. Ridley , T. B. Watkins , Proc. Phys. Soc. 1961, 78, 293.

[19] C. Hilsum , Proc. IRE 1962, 50, 185.

[20] H. Yasunaga , J. Phys. Soc. Jpn. 1968, 24, 1035.

[21] K. Rubi , J. Gosteau , R. Serra , K. Han , S. Zeng , Z. Huang , B. Warot‐Fonrose , R. Arras , E. Snoeck , M. G. Ariando , W. Escoffier , npj Quantum Mater. 2020, 5, 9.

[22] Z. Sroubek , Phys. Rev. B 1970, 2, 3170.

[23] A. H. Kahn , A. J. Leyendecker , Phys. Rev. 1964, 135, A1321.

[24] V. K. Guduru , A. Granados del Aguila , S. Wenderich , M. K. Kruize , A. McCollam , P. C. M. Christianen , U. Zeitler , A. Brinkman , G. Rijnders , H. Hilgenkamp , J. C. Maan , Appl. Phys. Lett. 2013, 102, 051604.

[25] E. Schöll , Nonlinear Spatio‐Temporal Dynamics and Chaos in Semiconductors, Cambridge University Press, Cambridge 2001.

[26] H. Kroemer , Proc. IEEE 1964, 52, 1736.

[27] B. K. Ridley , J. J. Crisp , F. Shishiyanu , J. Phys. C: Solid State Phys. 1972, 5, 187.

[28] B. K. Ridley , Br. J. Appl. Phys. 1966, 17, 595.

