# Supplementary material for: Photoinduced Negative Differential Resistivity and Gunn Oscillations in SrTiO3
Source: Adv Sci (Weinh). 2023 Oct 23;10(35):2306420. doi: 10.1002/advs.202306420 (PMC10724436; doi:10.1002/advs.202306420)
Supplement: Supplementary file 1 — Supporting Information [file ADVS-10-2306420-s001.pdf]

ADVANCED SCIENCE
Open Access

Supporting Information

for *Adv. Sci.*, DOI 10.1002/advs.202306420

Photoinduced Negative Differential Resistivity and Gunn Oscillations in SrTiO3

*Mehrzad Soleimany and Marin Alexe**

Photoinduced Negative Differential Resistivity and Gunn oscillations in SrTiO3

Mehrzad Soleimany[1,2] and Marin Alexe[1]*

Supporting Information

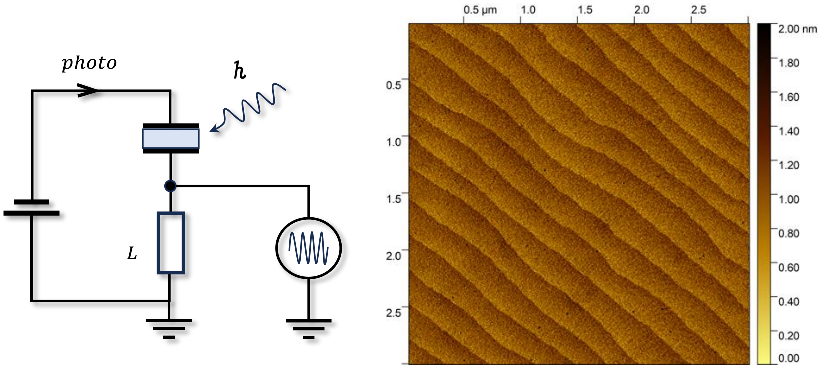

(a) **(b)**

Figure S1. **(a)** Circuit used for measuring the time-dependent photoconductivity. The load resistance R_L is 120 Ω. **(b)** Vicinal STO surface obtained after etching and annealing process. The step height is one unit cell.

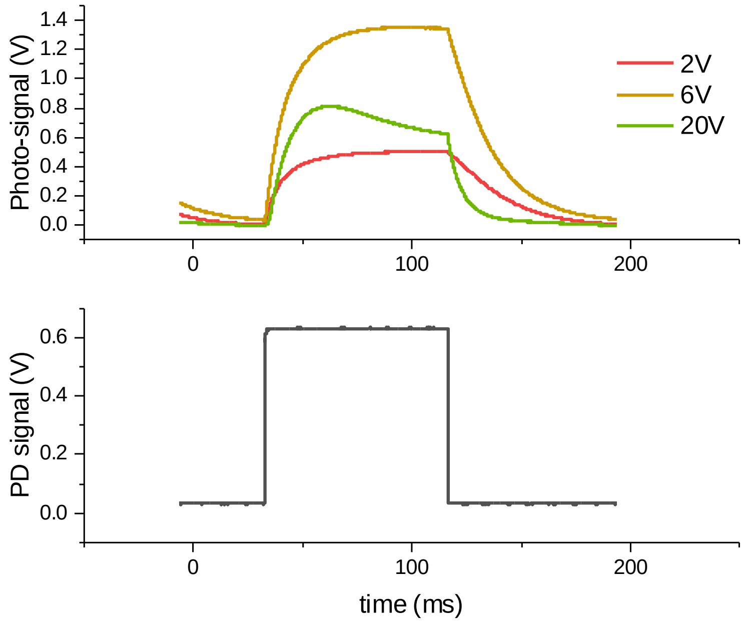

Figure S2. Time-dependent photocurrent (top) at different applied voltages measured at 10K by pulsing the light (375 nm) with 100 ms. The lower panel is the light waveform measured by a high-speed Si photodiode. The IV characteristics have been measured by varying the applied voltage and sampling the current in steady state, i.e., just before light cession. The time between the pulses was ~10x longer than the pulse width.

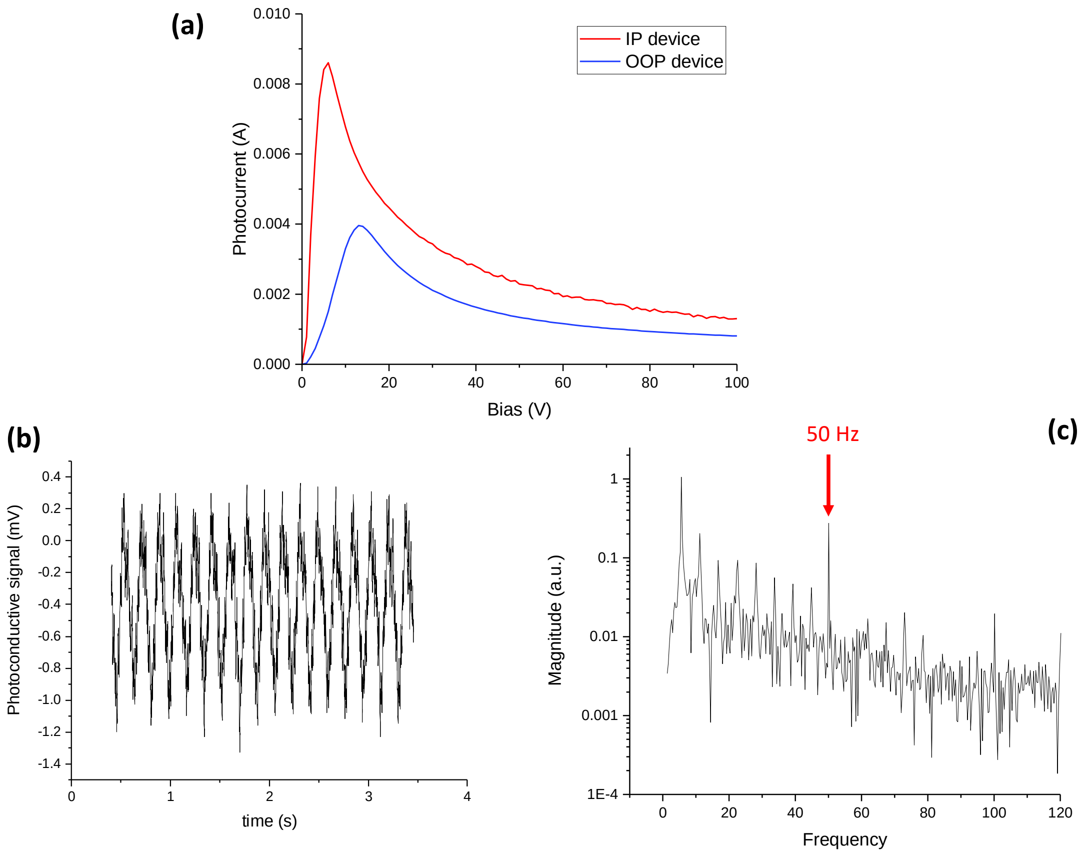

Figure S3. **(a)** IV characteristics measured for in both in-plane (IP) and out-of-plane (OOP) geometries at 10K under $\sim 8 \cdot 10^{13} cm^{-2} s^{-1}$ incident flux; **(b)** time dependence of the oscillating part of the IP photocurrent measured at 14K under illumination, using 375 nm LED with photon flux of about $10^{13} cm^{-2} s^{-1}$ and 50V applied voltage; **(c)** Fourier analysis of the oscillating photocurrent in (b) showing the fundamental frequency centred on about 5.5 Hz and the higher harmonics. The arrow indicates the 50Hz noise signal.

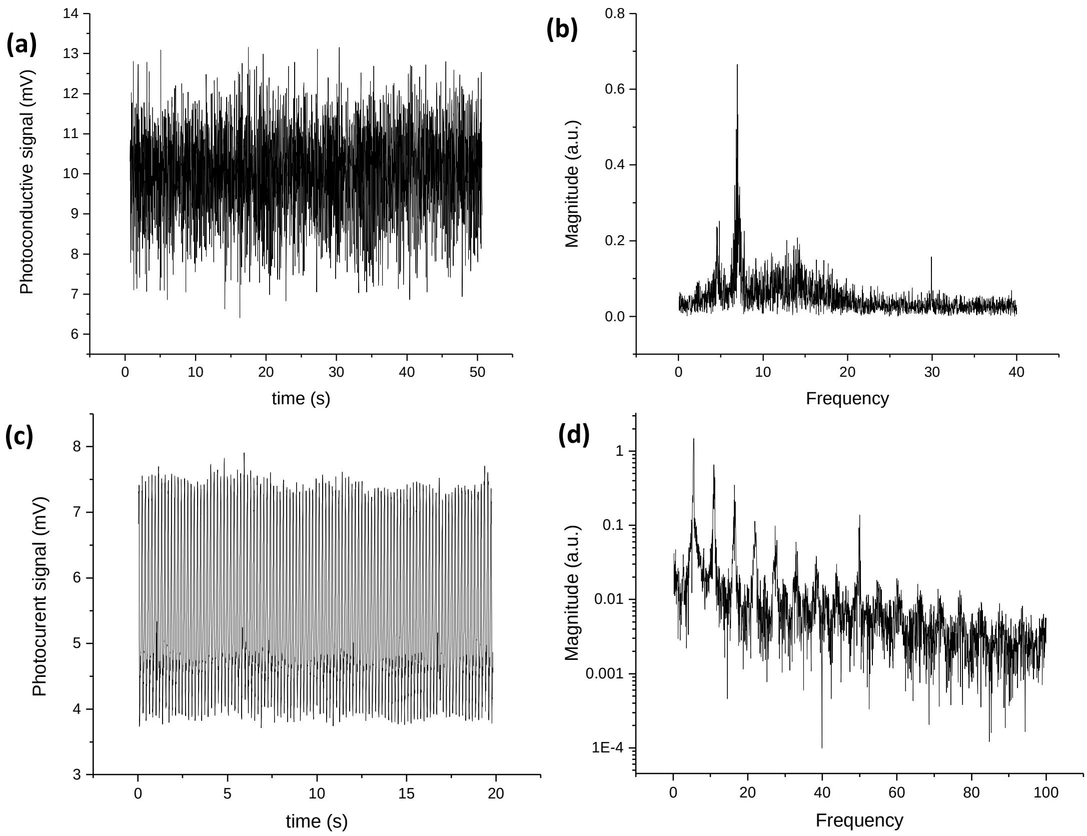

Figure S4. (a) Out-of-plane oscillations and (b) and the corresponding FFT analysis of a $SrTiO_3$ crystal roughly polished to 250µm; (c) Out-of-plane oscillations(b) and the corresponding FFT analysis of the $SrTiO_3$ crystal fine polished to 100µm. All measurements performed in similar conditions as in Fig. S3.
